# The role of Machiavellianism, cyberbullying, and family relationships in adolescent gaming addiction: a problem behavior theory approach

**DOI:** 10.3389/fpsyg.2026.1794127

**Published:** 2026-06-19

**Authors:** Enver Ulaş, Harun Ismail Incekara

**Affiliations:** 1 Istanbul Sabahattin Zaim Universitesi, İstanbul, Türkiye; 2 Department of Educational Sciences, Istanbul Medipol University, İstanbul, Türkiye

**Keywords:** adolescents, cyberbullying, gaming addiction, Machiavellianism, mediation, moderation, parent-adolescent relationship

## Abstract

**Background and objectives:**

This study investigates the relationship between gaming addiction and Machiavellianism in adolescents, focusing on the mediating role of cyberbullying and the moderating role of the parent–adolescent relationship.

**Methods:**

A total of 601 high school students in Istanbul participated in the study. Participants completed standardized measures assessing gaming addiction, Machiavellian traits, cyberbullying behaviors, and the quality of their relationship with their parents. Structural equation modeling (SEM) was used to test a moderated mediation model.

**Results:**

Cyberbullying significantly mediated the relationship between gaming addiction and Machiavellianism. Moreover, the parent–adolescent relationship moderated the link between gaming addiction and cyberbullying: the association was stronger among adolescents reporting lower relationship quality. Model fit indices indicated acceptable fit for the proposed model.

**Conclusion:**

These findings highlight the psychological and social pathways through which gaming addiction may relate to manipulative personality traits. Interventions aiming to improve family dynamics and reduce cyberbullying behaviors may help mitigate the negative effects of excessive gaming in adolescents.

## 1 Introduction

Digital games have become more visible in the daily lives of the young as technology advances. The widespread availability of internet access has accelerated this process. The limited availability of physical play spaces has also increased the turn toward digital gaming. Interest in games has grown particularly among adolescents. In parallel, the amount of time spent playing games has increased (Brooks et al., 2016; Jenkins, 2009). Recent research has increasingly examined the relationship between problematic gaming behaviors and online aggression among adolescents. Studies suggest that excessive engagement in digital gaming environments may be associated with a higher likelihood of online risk behaviors, including cyberbullying and other forms of digital aggression (Li et al., 2023; Kowalski et al., 2024). This expansion does not always lead to positive outcomes. Excessive and uncontrolled use of digital games may give rise to various problems. One of these is gaming addiction. Gaming addiction is often accompanied by difficulties in controlling play behavior. Over time, this pattern may interfere with daily functioning. It may also contribute to the development of unhealthy habits.

In research, problematic gaming has also been detailed using a number of other names. One common term is problematic gaming (Paquin et al., 2025; Stašek et al., 2025). Another common term is pathological video gaming (Choo et al., 2010). Some studies have also utilized the term internet gaming addiction (Kuss and Griffiths, 2012; Rosales-Navarro and Torres Pérez, 2025). In the DSM-5, the term Internet Gaming Disorder (IGD) is used (American Psychiatric Association [APA], 2013).

Regardless of the nomenclature used, there is consistent indication from research that too much involvement in digital gaming is harmful to quality of life. According to the DSM-5, IGD is described as: “A pattern of behavior in which there is difficulty in controlling online gaming, and this gaming may produce marked distress or impairment in social, occupational, or other important areas of functioning.” The DSM-5 further points out that “further research is needed before IGD or any similar disorder is considered a “mental disorder”” (American Psychiatric Association [APA], 2013).

The incidence of gaming addiction has increased worldwide. This situation may negatively affect the player socially and academically, with significant effects on the psychological wellbeing of gamers. Various researches have shown links between gaming addiction and depression, anxiety, stress, and social phobia (Loton et al., 2016; González-Bueso et al., 2018; Krossbakken et al., 2018; Wang et al., 2019).

The occurrence of Internet Gaming Disorder is not solely influenced by environmental variables, and personal traits of players are also important (Kuss and Griffiths, 2012; Şalvarlı and Griffiths, 2019; Gervasi et al., 2017). Certain studies indicate that personal variables like playtime and loneliness could play a more substantial role than interpersonal and contextual factors in developing a gaming disorder (Zhuang et al., 2023). This collectively points toward the fact that this issue of gaming addiction cannot be attributed to a single cause.

The Problem Behavior Theory (PBT) is a relevant conceptual paradigm in understanding multidimensional risk behaviors, such as gaming addiction (Jessor, 1991). PBT argues that risk behaviors during adolescence should not be attributed exclusively to the properties of the individual. Instead, risk behavior should be examined within the context of the intersection between social context, family, and culture. In PBT, the personal or psychological aspects of the systems that influence individual behavior relate to the value, expectancies, and psychological predispositions of the person (Jessor, 1991). The pivotal elements of the environmental or contextual aspects of the PBT framework involve family, school, and peer relations. In PBT, a balance between both perspectives or systems should be negotiated. The resiliency-enhancing aspects include family cohesion and supervision, while the risk-enhancing aspects include a low level of family support or low social control. The risk behavior of the adolescent would largely be a function of such a balance.

This model can also be very useful in the context of gaming addiction because it allows for the consideration of personal and environmental variables together. Accordingly, the theoretical base for the current study will rely on the PBT. This model has been employed very effectively in different cultural settings in several studies. This includes the demonstration of delinquent behaviors among Iranian adolescents (Darvishi et al., 2022) and various forms of problem behaviors among Irish youth (O’Connor et al., 2016). Likewise, it has also been employed effectively in various cultural settings for negative behavioral manifestations (Vazsonyi et al., 2008; Vazsonyi et al., 2010).

Research into gaming addiction has isolated a number of key personality traits common to people who display higher levels of gaming addiction. Among these is a reduction in self-esteem, as people with gaming addiction have reportedly lower levels of self-esteem (Savolainen et al., 2024). Raised levels of aggression and higher sensation-seeking have also become more common (Shahid et al., 2025; Taşbaşı et al., 2024).

Research also shows that gamers with gaming addiction have problems with negative emotional regulation and solving life problems (Kardefelt-Winther, 2014; Blasi et al., 2019). The motivational aspect of digital gaming increases, especially among those undergoing life stress. The support of family members is essential in this regard. Those adolescents with no family support and who face stress in their surroundings are especially at risk of adopting risky behaviors such as gaming addiction. Zhou and Xing (2021) make similar observations regarding gaming addiction and indicate that a lack of family support is correlated with increased levels of gaming addiction. Moreover, it is considered to be a protective element.

The Dark Triad consists of three interpersonal styles which have negative impacts on social behaviors, namely narcissism, Machiavellianism, and psychopathy (Paulhus and Williams, 2002). For the purpose of this study, Machiavellianism is perceived neither as a stable trait of personality nor as a personality disposition. On the contrary, Machiavellianism is perceived as a type of interpersonal style which might emerge and be strengthened through interpersonal interactions in adolescence. The online context is very supportive of such a process, thanks to its properties like anonymity, competition, and power strivings. Manipulative and self-centered behaviors might, in such a setting, become reinforced through time. Sanchez-Rabaza et al. (2023) confirmed a tight relationship between Machiavellianism and manipulatory behavior in cyber interactions.

In digital spaces that are prone to competitive and aggressive interactions in an ongoing manner, people may develop better-strategized ways of interacting with other people. Findings quoted in Curtis et al. in the year 2021 can attest to this. According to their study, digital spaces for playing games that involve competitive and control-oriented dimensions tend to have structural aspects that can favor and reward people who act manipulatively and selfishly. Moreover, aggressive and abusive online interactions found in problematic digital behaviors have also contributed to the development of control and manipulation-oriented tendencies in people (Goodboy and Martin, 2015; Calaresi et al., 2024 in the year 2024).

What is significant regarding gaming addiction is its link with cyberbullying. Cyberbullying is described as deliberate and repeated actions intended to cause harm via electronic devices such as computers and mobile phones, among others (Patchin and Hinduja, 2022).

Research has also shown that gamers who suffer from addiction have a higher chance of being both cyberbullying victims and cyberbullies (Andrea and Yuliati, 2019). This appears to be the case particularly where the gamer favors playing violent content games (Lam et al., 2013). A meta-analysis study carried out by Li et al. (2023) showed that people with a high level of addiction tend to have aggressive behavior tendencies more often. The study also pointed to the fact that people with a high level of aggressiveness are particularly susceptible to the risk of addiction, establishing a connection between the two.

In addition, adolescents preferring MMORPG type games have also shown to experience higher levels of gaming addiction as well as increased probabilities of experiencing symptoms for internet addiction (Stavropoulos et al., 2017; Smahel et al., 2008). Taking everything into consideration, it can be identified that the relationship between gaming addiction and cyberbullying forms a strong link that can have harmful outcomes for social and psychological wellbeing for adolescents.

Family relationship perception has a significant impact on behavioral concerns like gaming addiction and cyber bullying. It has been proven to have a direct impact on the use of the digital environment among youth. According to Colasante et al. (2022), European youth who have support from families tend to have low risks pertaining to gaming problems. Nielsen et al. (2020) also revealed correlations between problematic internet use, gaming addiction, negative parental practices, and unhealthy levels of family relations.

In some instances, students from dysfunctional families might be more prone to resorting to digital games. This behavior over time tends to create a conducive environment that leads to addiction. Based on this analysis, one of the important components that influence addiction is the nature of relationships within a family. This is supported by Schneider et al. (2017), who affirmed that dysfunctional family structures tend to increase improper gaming behavior. Indeed, during adolescence, addiction to gaming cannot solely depend on personal characteristics. Social issues that are associated with families play a crucial role within this process (Gerlach and Traxl, 2015; Keya et al., 2020; Tariq and Majeed, 2022).

From the existing literature, there are few studies that have explored the coexistence of Dark Triad traits, cyberbullying, and gaming addiction. Research carried out in the Turkish setting clearly indicates the research gap in this area. In fact, studies regarding family support and perceived relationship between parents and adolescents in the areas mentioned in the literature are quite limited (Çınar et al., 2021; Haylı, 2023). However, the finding in the Turkish setting generally corresponds with the finding mentioned in the international literature. However, these relationships are also shaped by cultural context. For this reason, findings should be interpreted with attention to local dynamics (Aytaç et al., 2022; Çimen, 2018; Colasante et al., 2022; Schneider et al., 2017). In this regard, further studies conducted in diverse cultural settings may help improve the generalizability of existing findings.

### 1.1 The present study

The primary aim of this study is to examine the relationship between gaming addiction and cyberbullying behaviors. The study approaches this relationship within the context of Machiavellian tendencies and perceived parent–adolescent relationships. Accordingly, it is assumed that cyberbullying may function as a mediating factor in the association between problematic gaming and Machiavellian tendencies. In addition, the quality of parent–adolescent relationships is expected to moderate the relationship between gaming addiction and cyberbullying.

Previous research has shown that personality characteristics such as Machiavellianism and problematic online behaviors are meaningfully associated with gaming addiction (Gervasi et al., 2017; Andrea and Yuliati, 2019). However, the role of environmental protective factors such as family support has been examined only to a limited extent, particularly as a moderating variable. To the authors’ knowledge, Dark Triad personality traits, cyberbullying behaviors, and gaming addiction have not previously been tested together within a comprehensive model that incorporates family relationships.

**FIGURE 1:**
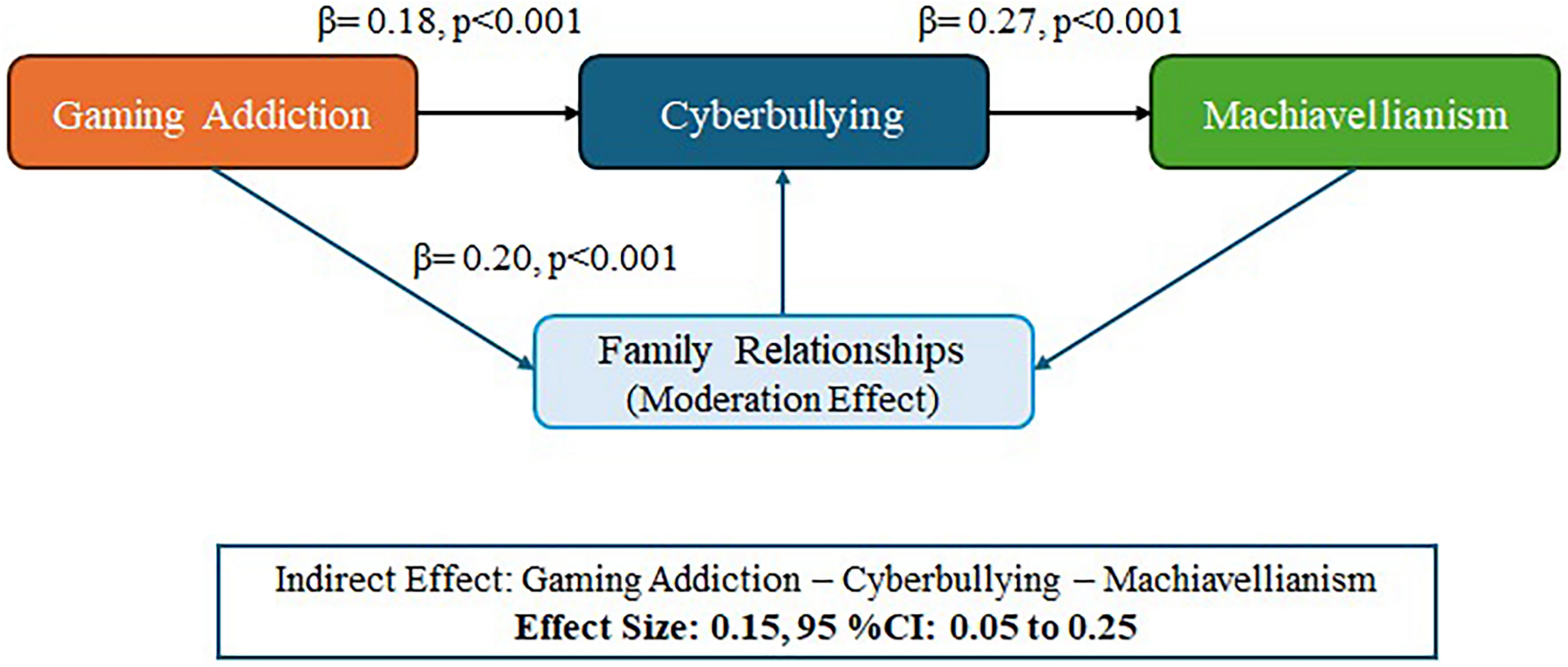
Moderated mediation model of the relationship between gaming addiction and Machiavellianism. Standardized path coefficients are shown. Indirect effect = 0.15, 95% CI [0.05, 0.25]. All paths are statistically significant at *p* < 0.01.

Within the framework of Problem Behavior Theory (Jessor, 1991), the present study assumes that gaming addiction may increase cyberbullying behaviors, and that cyberbullying may, in turn, be associated with Machiavellian tendencies. It is further assumed that the quality of perceived parent–adolescent relationships moderates the association between gaming addiction and cyberbullying. Based on these assumptions, a model was tested in which cyberbullying functions as a mediator and parent–adolescent relationships function as a moderator. The hypothesized model is presented in Figure 1.

*H1:* Gaming addiction positively predicts cyberbullying behaviors among adolescents.

*H2:* Cyberbullying behaviors are positively associated with Machiavellian tendencies among adolescents.

*H3:* Cyberbullying mediates the relationship between gaming addiction and Machiavellian tendencies.

*H4:* The quality of perceived parent–adolescent relationships moderates the association between gaming addiction and cyberbullying, such that stronger parent–adolescent relationships weaken this association.

## 2 Methodology

### 2.1 Participants and procedure

This study was conducted to examine gaming addiction among high school students in Turkey. According to data from the Millî Eğitim Bakanlığı (2025), the total population of high school students consists of 5,796,881 individuals. Based on a 95% confidence level and a 5% margin of error, Krejcie and Morgan’s (1970) sample size table indicated that a minimum of 384 participants would be sufficient. Within the context of this study, data was sought from 601 students in total.

Participants were recruited through school counselors from eight districts of Istanbul (Fatih, Bakırköy, Bahçelievler, Bağcılar, Esenyurt, Küçükçekmece, Beylikdüzü, and Büyükçekmece). A convenience-based recruitment strategy was used due to accessibility through collaborating schools. Participation was voluntary and open to all students who agreed to participate. Therefore, the sample should be interpreted as a non-probability sample and may not be fully representative of all Turkish adolescents.

Data collection involved online usage of Google Forms following parental consent. The period used for data collection went for approximately 4 weeks, and it took 10–15 min for respondents to complete on their own. To evaluate the clarity of measurement instruments used, a pilot study involved five additional students and did not form part of the study.

The purpose of using email verification was to ensure that all participants submitted feedback only once. The links were distributed via WhatsApp by a collaborating researcher alongside the school counselors. It was a voluntary process where the participants were not rewarded for their feedback. The researcher’s contact was submitted for student inquiries once the data was collected.

### 2.2 Data collection instruments

#### 2.2.1 Demographic information form

The demographic information sheet was created by the researcher to determine personal information about participants. This sheet consisted of inquiries regarding gender, age, parental educational attainment, the gaming platform used most often, categories of games played, and average number of gaming hours in a day.

#### 2.2.2 Gaming addiction scale for adolescents—short form

For measuring digital gaming addiction among adolescents, the scale devised by Anlı and Taş (2018) has been used for analysis. This scale consists of nine items and has a single-factor structure. The scale uses a 5-point Likert-type scale ranging from Never to Always. From factor analysis, it has been revealed that one factor accounted for 42.806% variance. The item factor values range from 0.437 to 0.761. The inter-item total correlation values range from 0.340 to 0.653, and communalities range from 0.191 to 0.579. This shows a moderate degree of inter-item correlation. Cronbach alpha value for measuring internal consistency for the original validation process reported 0.81, indicating adequate scale consistency. Cronbach alpha for analysis has been calculated at 0.87. The scale scores are positively related to digital gaming addiction among adolescents.

#### 2.2.3 Cyberbullying scale

In the conducted study, the Cyber Bullying Scale prepared by Arıcak et al. (2012) was used. The scale has a total of 24 items and uses a four-point response option. Responders answered each question using Never, Sometimes, Often, and Always. Scoring starts from 1 (Never) to 4 (Always), and the scores range from a total of 24 to a total of 96. This scale does not use reverse coding. The scores are higher when there are higher levels of cyberbullying. In the validation study, the internal consistency reliability using Cronbach’s alpha was found to be 0.95, and the test-re-test reliability using Pearson’s rho was found to be 0.70 for *n* = 103 participants. Construct validity was tested using exploratory factor analysis and found to have one component that explained 50.58% of the variability. The correlations varied between 0.49 and 0.80. In this study, the internal consistency reliability using Cronbach’s alpha was found to be 0.89.

#### 2.2.4 Parent–adolescent relationship scale

In the present research, the scale created by Aktaş (2017) was utilized. This scale includes a total of 27 questions and a five-choice format for responses: from Strongly Disagree to Strongly Agree. It takes scores from 1 (Strongly Disagree) to 5 (Strongly Agree), and total scores range from 27 to 135. It includes four sub-scales: support, sharing, closeness, and monitoring. In the original research, the Cronbach alpha of the total scale was found to be 0.95. Split-half reliability was also calculated as *r* = 0.97. In the present research, the Cronbach alpha of the total scale was found to be 0.91.

#### 2.2.5 Machiavellianism scale

This study also made use of Machiavellianism Scale originally developed for use with Children and Adolescents by Christie and Geis (1970), adapted to Turkish language and structure by Bayraktar (2021). This test has a total of 20 items and a five-point response format ranging from Strongly Disagree (1) to Strongly Agree (5). The test has three subscales with Cynicism toward Human Nature, Deceitful Behavior, and Distrust. The reverse scored Machiavellianism items have numbers 2, 4, 6, 9, 10, 11, 14, 16, 17, and 19. This study made use of this test to see to what extent adolescents act in a manipulative and self-centered manner in interpersonal relationships. This study gave this test an internal consistency coefficient of 0.82, and its Cronbach alpha was 0.84.

### 2.3 Data analysis

Analysis of data was performed using SPSS 22.0 and AMOS 24. Confirmatory Factor Analysis (CFA) was used to evaluate the fit of the measurement part of the model, where evaluation of item loadings and constructs and validity were considered (Bagozzi and Yi, 1988; Brown, 2015). Structural Equation Modeling (SEM) was applied to test the hypothetical model. Fit criteria utilized were χ^2^/df, CFI, TLI, SRMR, and RMSEA (Kline, 2023).

Within this context, the moderating function of perceived parent-adolescent relationship on the relationship between gaming addiction and cyberbullying was tested. Indirect effects were estimated by the bootstrapping method with resampling size of 5,000, and the cutoff for significance being 95% confidence intervals. All mediation analyses were performed following modern methodological guidelines employing the bootstrapping technique.

## 3 Results

In Table 1, the descriptive statistics and the internal consistency coefficients (cronbach alpha values) for the variables utilized within the study are indicated. The values exceeded 0.80, indicating strong internal consistency for all the scales utilized within the study. The skewness and kurtosis values were within the ± 1 limits, and these indicate that the variables met the assumptions of normal distribution.

**TABLE 1:** Descriptive statistics and Cronbach’s alpha results.

Variable	Mean (M)	Standard deviation (SD)	Skewness	Kurtosis	Cronbach’s alpha (α)
Gaming addiction	3.50	0.90	0.05	−0.10	0.87
Cyberbullying	3.10	0.85	0.10	−0.30	0.89
Parent–adolescent relationships	3.40	0.62	−0.15	−0.20	0.91
Machiavellianism	3.05	0.70	0.02	−0.25	0.84

Higher scores indicate greater levels of each construct. Cronbach’s alpha values above 0.70 indicate acceptable internal consistency.

All correlations were tested using two-tailed analyses at the 95% confidence level.

*H1*: Structural equation modeling results indicated that gaming addiction positively and significantly predicted cyberbullying behaviors (β = 0.18, *p* = 0.001), supporting H1.

*H2*: Cyberbullying behaviors significantly predicted Machiavellian tendencies (β = 0.27, *p* < 0.001), supporting H2.

*H3*: The findings show that cyberbullying mediates between gaming addiction and Machiavellianism. Though the correlation between gaming addiction and Machiavellianism was significant (*r* = 0.35, *p* < 0.05), due to a higher correlation between cyberbullying and Machiavellianism (*r* = 0.48, *p* < 0.001), the hypothesis of mediation proves true and hence confirms H3.

*H4:* The perceived parent to adolescent relationship had a significant moderating effect on the relationship between gaming addiction and cyberbullying. The positive relationship between the parent to adolescent relationship and gaming addiction was significant (*r* = 0.28, *p* < 0.05), and there was a significant negative relationship between the parent to adolescent relationship and cyberbullying (*r* = -0.32, *p* < 0.001).

The results from Confirmatory Factor Analysis (CFA) show that all the loadings are >0.70, thus verifying the structural representativeness of the model. In this regard, the Gaming Addiction (λ = 0.83), Cyberbullying (λ = 0.79), Parent-Adolescent Relationships (λ = 0.86), and Machiavellianism (λ = 0.82) have very high contributions to the model, which are attested to by the loadings. *t*-values for all the constructs range from 8.60 to 9.25 and are significant at *p* < 0.001. This suggests that all the variables have made significant contributions to the model and the measures are reliable. In addition, the measurement model demonstrated acceptable model fit according to commonly recommended thresholds. The fit indices indicated a satisfactory fit (χ^2^/df = 2.02, CFI = 0.912, TLI = 0.894, SRMR = 1.042, RMSEA = 0.040), suggesting that the measurement structure adequately represented the observed data.

The fit indices of the model are within acceptable limits. The chi-square to degrees of freedom ratio is below the recommended threshold. The CFI value of 0.912 and the TLI value of 0.894 indicate acceptable model fit. The SRMR value of 0.042 meets the recommended threshold and indicates good model fit. The RMSEA value of 0.040 also suggests a good fit of the model.

It should be noted that the standardized path coefficients obtained from the structural equation model represent relationships between variables while controlling for other variables included in the model. Therefore, these coefficients may differ from the bivariate correlations reported in Table 2. The smaller path coefficient (β = 0.18) compared to the correlation (*r* = 0.42) reflects the shared variance among the predictors and the multivariate nature of the SEM model rather than a statistical inconsistency. The confirmatory factor analysis results and model fit indices are presented in Tables 3, 4, respectively.

**TABLE 2:** Correlation matrix.

	Gaming addiction	Cyber- bullying	Machia- vellianism	Parent-adolescent relationships
Gaming addiction	1.00	0.42**	0.35*	0.28*
Cyberbullying	0.42**	1.00	0.48**	−0.32**
Machiavellianism	0.35*	0.48**	1.00	−0.29*
Parent–adolescent relationships	0.28*	−0.32**	−0.29*	1.00

*p < 0.05,

**p < 0.001.

**TABLE 3:** Confirmatory factor analysis results.

Variable	Factor loading (λ)	Error (ε)	*T*-value
Gaming addiction	0.83	0.29	8.95
Cyberbullying	0.79	0.33	8.60
Machiavellianism	0.82	0.30	8.75
Parent–adolescent relationships	0.86	0.25	9.25

All factor loadings are standardized and statistically significant at *p* < 0.001.

**TABLE 4:** Model fit indices.

Model fit indices	χ ^2^/df	CFI	TLI	SRMR	RMSEA
	2.02	0.912	0.894	0.042	0.040

Path analysis results presented in Table 5 indicate that gaming addiction positively and significantly predicts cyberbullying behaviors (β = 0.18, *p* = 0.001). In addition, cyberbullying behaviors were found to positively and significantly predict Machiavellian tendencies among adolescents (β = 0.27, *p* < 0.001).

**TABLE 5:** Acceptable model fit is indicated by χ^2^/df < 3, CFI and TLI ≥ 0.90, SRMR ≤ 0.08, and RMSEA ≤ 0.06. Standardized path coefficients of the structural equation model.

Path	Standardized BETA (β)	SE	*T*-value	*P*-value	Significance
Gaming addiction → cyberbullying	0.18**	0.05	3.40	0.001	Significance
Cyberbullying → machiavellianism	0.27**	0.06	4.50	< 0.001	Significance
Family relationships × gaming addiction → cyberbullying (moderation effect)	0.20**	0.05	3.30	0.001	Significance

*P* < 0.05;

*p < 0.01;

**p < 0.001.

Moreover, the interaction term between perceived parent–adolescent relationships and gaming addiction showed a significant moderating effect on cyberbullying (β = 0.20, *p* = 0.001). These results demonstrate that both the direct and moderating effects tested in the model were statistically significant. The mediating role of cyberbullying in the relationship between gaming addiction and Machiavellian tendencies was examined using bootstrapping procedures, and the results are presented in Table 6.

**TABLE 6:** Bootstrapping results for mediation analysis.

Effect type	Effect size	Lower CI	Upper CI
Direct effect	0.20	0.08	0.31
Indirect effect	0.15	0.05	0.25
Total effect	0.35	0.22	0.48

Bootstrapping analysis with 5,000 resamples was conducted to examine the mediating role of cyberbullying in the relationship between gaming addiction and Machiavellian tendencies. The indirect effect was statistically significant [β = 0.15, 95% CI (0.05, 0.25)], as the confidence interval did not include zero. The direct effect of gaming addiction on Machiavellianism remained significant (β = 0.20), indicating partial mediation.

The proposed moderated mediation model is shown in Figure 1. Gaming addiction significantly predicted cyberbullying behavior (β = 0.18, *p* = 0.001), which in turn predicted Machiavellian tendencies (β = 0.27, *p* < 0.001). Cyberbullying significantly mediated the relationship between gaming addiction and Machiavellianism [indirect effect = 0.15, 95% CI (0.05, 0.25)]. In addition, family relationships moderated the link between gaming addiction and cyberbullying (β = 0.20, *p* = 0.001), indicating that this association varied depending on the quality of parent–adolescent relationships.

## 4 Discussion

This study examined the relationships between gaming addiction and Machiavellian behaviors and cyberbullying in a complex framework that encompasses family relations as well. The findings from the analysis were supported for all hypotheses that existed within this study. The implication here is that gaming addiction is a process that cannot be sufficiently understood if one focuses on individual factors alone.

When the findings are juxtaposed with the lens provided by the Problem Behavior Theory, they clearly highlight that the findings point to the pivotal role that the social context plays in contributing to the manifestations of addictive behavior in the digital world. Taken together, the findings clearly point to the fact that the addiction of gaming is a phenomenon that goes beyond the realms of the trait level.

Consistent with the first hypothesis, a positive and statistically significant correlation was found between gaming addiction and cyberbullying behavior. The result indicates that greater involvement in online gaming activities and a higher degree of addiction are potentially correlated with a greater tendency for aggressive behavior online. Increasing literature has supported the correlation between intensive online gaming engagement and cyberbullying (Kowalski et al., 2019; Kowalski et al., 2014; Li et al., 2023; Yang et al., 2025; McInroy and Mishna, 2017; Cotler et al., 2017).

Previous research has suggested that video games with violence could normalize behaviors such as aggression, dominance, and punishment and might be applicable to online social interactions (Anderson and Bushman, 2018; Velez et al., 2016; Li et al., 2023). This can be further encouraged through online platforms for games characterized by social interactions and anonymity (Yang et al., 2025; Cotler et al., 2017; McInroy and Mishna, 2017). As suggested, behaviors related to cyberbullying have been identified to be increasingly observed through interactions performed with social media platforms and online games (Kowalski et al., 2024).

At the same time, evidence suggests that environmental factors such as family support and parental monitoring may reduce adolescents’ likelihood of engaging in risky online behaviors (Buelga et al., 2016). When interpreted through the lens of Problem Behavior Theory, these findings form a coherent pattern. According to the theory, risky behaviors emerge through the interaction between individual tendencies and environmental conditions (Jessor, 1991). From this perspective, gaming addiction can be viewed as a risk factor that facilitates aggressive behaviors in online settings. Importantly, gaming addiction appears to be shaped not only by individual processes but also by adolescents’ engagement with digital culture.

With regard to the second hypothesis, a significant positive relationship was observed for cyberbullying behaviors and Machiavellianism. This suggests that people who score high on Machiavellianism have a propensity for undesirable behavior within cyberspace. Competitive and dominance-based game settings may make such propensities more evident. Other factors that might make such propensities evident include anonymity and a perception of control in cyberspace, where people with such Machiavellian propensities tend to have no compunctions about crossing social boundaries.

The findings correlate well to existing studies. Jones and Paulhus (2009), for instance, were able to connect Machiavellianism to manipulative and strategic tactics. On the other hand, the study by Curtis et al. (2021) found that the same characteristics may have benefits within online gaming communities. In a similar way, the studies of Calaresi et al. (2024) and Xu et al. (2023) found out that the Machiavellian tendencies of online gamers are often observed to be manifest as the attainment of strategic superiority, the manipulation of players, as well as occasional aggressive behavior. On the other hand, the studies performed by Leite et al. (2023) suggest a different perspective on this issue.

When these traits are considered in the context of recurrent gaming interactions, problematic behavioral tendencies could be observable and get reinforced. Under the framework of the Problem Behavior Theory, it is not entirely unexpected that this is the case because the theory asserts the potential for personal characteristics to be expressed in the form of problematic behaviors when opportunity is the guiding consideration. In line with this stance, it may be deduced from the results that Machiavellianism represents an interpersonal style that could be developed and reinforced using computer-based interactions.

As far as the third hypothesis is concerned, it has been seen that cyberbullying acts as a mediator between gaming addiction and the development of Machiavellian attitudes. This result tends to show that the higher the level of gaming addiction, the greater the chances or the level of cyberbullying behaviors. Over time, this type of risky behavior may also act as the initiating cause of the enhancement of the level of Machiavellian attitudes.

In such a context, cyberbullying cannot only represent a consequence of overgaming but can as well mean a habitual form of social experience which upholds manipulation- and dominance-oriented patterns of interaction. In such a way, cyberbullying represents a kind of context where some interpersonal dispositions can be actualized.

Current results are in line with previous studies. For instance, it has been shown by Kircaburun et al. (2018) and Lee et al. (2024) that aggressive behaviors in gaming environments are intricately interwoven with manipulative behaviors, which may lead to addictive behavior. In a similar vein, studies conducted by Demircioğlu and Göncü-Köse (2023) and Calaresi et al. (2024), have shown cyberbullying to be a mediating variable between gaming addiction and Machiavellianism. This is in line with what is stipulated in Problems Behavior Theory, whereby behaviors are seen to emerge due to a conjoined outcome of internal predispositions combined with environmental interactions (Jessor, 1991).

Regarding the fourth hypothesis, parent and adolescent relationship variables were discovered to have a moderating effect on the relationship between gaming addiction and cyberbullying. This suggests that as levels of gaming addiction increase, so do cyberbullying behaviors among adolescents with weak family ties. This can be diminished with strong parent and adolescent relationship variables.

The positive interaction coefficient does not contradict the negative correlation observed between parent–adolescent relationship quality and cyberbullying. Correlations represent direct bivariate associations, whereas moderation coefficients reflect interaction effects. The moderation result indicates that the strength of the association between gaming addiction and cyberbullying varies depending on the level of parent–adolescent relationship quality. In particular, weaker parent–adolescent relationships appear to strengthen the link between gaming addiction and cyberbullying.

The results imply that family relations could have a protective role concerning risky online conduct. A strengthening of the parent-teen relationship means that the likelihood of gaming addiction being transformed into cyberbullying will be lowered. In this regard, the family may act more as an environmental regulator of behavioral predispositions instead of an actual control agent. These findings also highlight the importance of incorporating family-based components into prevention and intervention programs addressing digital risk behaviors among adolescents. Strengthening parent–adolescent communication and parental monitoring may help reduce adolescents’ involvement in problematic online activities.

The current findings support previous ones. For instance, Liang et al. (2021) found correlations between dysfunctional family structures and greater levels of digital risk behaviors. Similarly, Feng et al. (2023) and Alvarez-Subiela et al. (2022) confirmed the moderating effect of family support in preventing gaming addiction. Furthermore, Tsankova et al. (2024) proved that the relationship between cyberbullying and problematic internet use is moderated by parent and child relationships. Notably, this moderation effect cannot be termed as prevention in its entirety; it only shows how these relationships have the potential to reduce risk levels in dangerous digital behaviors.

The results of the research indicate that the relationships between gaming addiction, Machiavellian behavior, and cyberbullying cannot be attributed to the variables of the participants. The context in which the participants live seems to influence the correlations. In the Turkish context, the online world that the adolescents live in is a place where they find themselves at the same time facing school pressure, economic instability, and stress. The online world becomes a refuge for the youths.

More competitive processes, like those undertaken for entering university examinations, might as well push these youth to pursue experiences of success, control, or power within online platforms. The situation after COVID-19 has unveiled these mechanisms due to accelerated digitalization as well as a decrease in face-to-face communication. Studying these mechanisms within a specific context of Turkish youth seems particularly meaningful considering the strong combination of competitiveness in academic environments, a large degree of family interdependence, as well as strongly accelerated digitalization of these youth. Social skills problems might as well increase these youth’s tendencies for violating boundaries online. These tendencies may appear especially for those youth who have stronger Machiavellian dispositions.

The anonymity that comes with the digital environment is another facilitator aspect that helps increase the potential for the emergence of these behaviors. It is from this perspective that the application of individual psychological aspects as the only remedy in the prevention and intervention for digital risk behaviors seems insufficient.

While the focus of much literature to date has been on the role of risk factors at the individual level, it is proposed that the perceived relationships between parent and adolescent in the current study act as a boundary condition that affects the impact of gaming addiction on cyberbullying and maladaptive social behavior.

Based on the findings of the present study, where gaming addiction was found to significantly predict cyberbullying, which in turn mediated the development of Machiavellian tendencies—and where perceived parent–adolescent relationship moderated this pathway—there are several implications for clinical practice and future research.

Interventions for adolescents exhibiting problematic gaming behavior should target not only individual behavioral symptoms but also interpersonal and cognitive patterns, including manipulative tendencies and online aggression.

Since perceived parent–adolescent relationship was found to buffer the link between gaming addiction and cyberbullying, parental involvement in intervention programs appears essential. Psychoeducational programs can enhance parental monitoring, emotional closeness, and communication skills.

Cognitive Behavioral Therapy (CBT) modules tailored for online aggression and impulsivity may help reduce both gaming addiction and Machiavellian coping strategies. Social skills training could also reduce reliance on manipulative behaviors in online settings.

School counselors and psychologists could be trained to recognize early signs of risky online behaviors and initiate support programs focusing on self-regulation and empathy-building.

Future research could examine whether the observed mediation and moderation effects remain stable over time, and whether intervention on family dynamics could causally reduce digital aggression and manipulative behavior.

Considering the role of cultural norms in shaping family dynamics and adolescent behavior, similar models should be tested in different sociocultural settings.

Investigating whether gender moderates these relationships may provide more tailored implications for intervention.

Several limitations should be considered when interpreting the findings of this study.

First, due to the cross-sectional design, no causal conclusions can be drawn regarding the relationships between gaming addiction, cyberbullying, Machiavellian traits, and family relationships. Future studies adopting longitudinal or experimental designs would be better suited to explore the directionality and temporal stability of these associations.

Second, the study relied solely on self-report questionnaires, which are susceptible to social desirability bias and recall inaccuracies. Although validated instruments were used, it is possible that participants underreported or overreported their behaviors and attitudes.

Third, the sample was recruited from eight districts within a single metropolitan area in Turkey, using a convenience sampling method, which limits the generalizability of the results to broader populations. Adolescents from different regions or cultural contexts may display different behavioral patterns and family dynamics. Additionally, the use of a non-probability sampling strategy may limit the generalizability of the findings to the broader population of Turkish adolescents.

Fourth, while the study incorporated a psychosocial model (PBT), other relevant psychological or environmental variables—such as impulsivity, peer influence, or school climate—were not measured. Including such factors in future research would provide a more comprehensive picture of adolescent risk behaviors.

Finally, although Machiavellianism was conceptualized as an emerging interpersonal style, the study did not investigate the long-term development or contextual reinforcement of such traits. Qualitative or mixed-method studies could help explore how adolescents make sense of their manipulative behaviors in digital environments.

## 5 Conclusion

The current study shows a positive correlation between gaming addiction and online activities of adolescents. That is, as a level of gaming addiction increases, so does a rate of cyberbullying. Conversely, cyberbully seems to influence a greater development of Machiavellian characteristics over a period of time. The current findings propose that a view of gaming addiction from a merely personal perspective might be inappropriate; instead, it can be assumed as a tendency to problematic behavior expressed online.

The second major finding is related to the importance of the role of family relationships. Compared to those who experience weaker parent-adolescent relationships, the correlation between gaming addiction and cyberbullying tends to be weaker among those who experience stronger parent-adolescent relationships. This finding suggests that family relationships help to buffer the negative effects of digital risk behaviors among the adolescents.

Cumulatively, the above findings suggest that viewing behavioral addiction to games as purely a matter of individual personality factors is not adequate. To better understand the behavior of adolescents on-line, one has to take into account factors of family relations and the broader social and contextual framework within which such behavior takes place. This study could provide some leads for further investigation on-lines on how such dynamics might play out over time and if similar associations hold.

## Data Availability

The datasets presented in this article are not readily available because The datasets generated during and/or analyzed during the current study are not publicly available due to ethical restrictions and participant confidentiality. Requests to access the datasets should be directed to Harun Ismail Incekara, incekara-harun@outlook.com.tr.
